# Internal Nano Voids in Yttria-Stabilised Zirconia (YSZ) Powder

**DOI:** 10.3390/ma10121440

**Published:** 2017-12-18

**Authors:** Chen Barad, Gal Shekel, Michael Shandalov, Hagay Hayun, Giora Kimmel, Dror Shamir, Yaniv Gelbstein

**Affiliations:** 1 The Unit of Energy Engineering, Ben-Gurion University of the Negev, Beer-Sheva 84105, Israel; chenhu@post.bgu.ac.il; 2 Department of Materials Engineering, Ben-Gurion University of the Negev, Beer-Sheva 84105, Israel; shekel.gal@gmail.com (G.S.); hagayha@post.bgu.ac.il (H.H.); 3 NRCN, P.O. Box 9001, Beer-Sheva 84190, Israel; michash1234@gmail.com (M.S.); drorshamir@gmail.com (D.S.); 4 Institutes for Applied Research, Ben-Gurion University of the Negev, Beer-Sheva 84105, Israel; gyorakimmel@gmail.com

**Keywords:** 7–8 mol % YSZ, sol-gel, freeze-dry, XRD Rietveld refinement, internal nano-voids

## Abstract

Porous yttria-stabilised zirconia ceramics have been gaining popularity throughout the years in various fields, such as energy, environment, medicine, etc. Although yttria-stabilised zirconia is a well-studied material, voided yttria-stabilised zirconia powder particles have not been demonstrated yet, and might play an important role in future technology developments. A sol-gel synthesis accompanied by a freeze-drying process is currently being proposed as a method of obtaining sponge-like nano morphology of embedded faceted voids inside yttria-stabilised zirconia particles. The results rely on a freeze-drying stage as an effective and simple method for generating nano-voided yttria-stabilised zirconia particles without the use of template-assisted additives.

## 1. Introduction

Pure zirconia (ZrO_2_) undergoes a monoclinic (*P*2_1_/*c*) → tetragonal (*P*42/*nmc*) → cubic (*Fm*3*m*) phase transition sequence upon heating [1]. The large volume expansion associated with the transition between the tetragonal and monoclinic phases upon cooling prevents exploitation of the refractory properties of pure zirconia as a structural ceramic. This disruptive phase transition can be suppressed, and even totally avoided, by stabilising the tetragonal or the cubic phases upon doping with yttria (Y_2_O_3_), depending on its molecular fraction, for obtaining yttria-stabilised zirconia (YSZ, or ZrO_2_-Y_2_O_3_) compositions. It is known that the tetragonal phase is more mechanically favourable for practical applications than the cubic phase, and yet improving mechanical properties of YSZ-based materials still remains a technology challenge especially for Solid Oxide Fuel Cell (SOFC) electrolytes and medical and dental implants [2]. 7–8 YSZ (7–8 mol % Y_2_O_3_ doping of ZrO_2_) is the most popular investigated compositional range of YSZ used for numerous applications [3]. Therefore, this was selected for this study.

Porous YSZ-based materials are gaining more importance and popularity nowadays, due to a variety of applications. For instance, in the energy field, as SOFC supports and thermal insulators; in environmental use as ceramic filters and as electrochemical sensors of monitoring NO*_x_* [4].

Plasma spray and electron beam deposition are both key processing methods applied to deposit YSZ coatings for Thermal Barrier Coating (TBC) uses. Hollow spherical YSZ powders are broadly in use as the main feedstock powder for plasma sprayed coating [5,6,7]. The unique morphology of hollow spheres is known to reduce thermal conductivity significantly, relative to other feedstock materials, and that the thermal properties of the final coatings are directly influenced by the internal particle’s morphology of voids in initial powder [8]. Different spray pyrolysis (SP) techniques have been technologically advanced to produce ceramic powders. The SP techniques are composed of many stages and consume large amounts of fuel gas, such that modified SP techniques are being researched [9,10]. Hollow YSZ particles are also under research regarding the synthesis of NiO-YSZ composite particles for an electrode of SOFC by spray pyrolysis [11].

Considering the thermoelectric approach, the thermal conductivity (κ) of air as in voids (0.024 W/(m·K)), is about two orders of magnitude lower than for YSZ (2.2–2.9 W/(m·K)) [12]. Since YSZ is an electronic insulator, the main contribution to the thermal conductivity in this material class is predominantly attributed to lattice vibrations (phonons), κ_l_ [13]. It is well known that nano-features act as effective phonon-scattering centres, decreasing κ_l_. Therefore, for thermal-barrier applications or dental implants, a significant reduction of the thermal conductivity in YSZ is expected upon generation of nano-featured compositions, containing air-voids, as a secondary thermally insulating phase. These two contributions might be combined by the generation of nano-voids in YSZ based materials.

Ice templated porous alumina structures have been reported as a technique leading to numerous morphological features in which pores were a replica of the origin ice crystals [14]. Facetted internal voids were also achieved in alpha-alumina particles via a ball-milling process [15]. Regarding YSZ-based materials, aligned pore channels were prepared by freezing a slurry of YSZ and tert-butyl alcohol (TBA) additive, followed by removing the frozen TBA in vacuum [16]. Porous YSZ using freeze-casting techniques with the addition of polyvinyl alcohol (PVA) in a ball-milling process resulted in lamellar pores [17]. As far as we know, nano-faceted voided YSZ powder particles prepared by sol-gel technique followed by freeze-drying process (without the usage of additives) has never been shown.

In the current research, we propose a procedure for the synthesis of 7–8 YSZ with embedded nano-voids by the sol-gel technique followed by the freeze-drying approach.

The obtained morphology was investigated via transmission electron microscopy (TEM) while the phase analysis was performed using X-ray diffraction (XRD). Crystal size and void size were approximated based on the measurement of more than 150 well-defined crystals/voids in TEM images using *ImageJ* (image processing software). Statistical analysis was performed using statistical package for the social sciences (SPSS) software.

## 2. Materials and Methods 

7 YSZ and 8 YSZ powder batches were synthesised via the sol-gel method reported in [18], using zirconyl chloride octahydrate (ZrOCl_2_·8H_2_O) (purity ~99.5%) and yttrium nitrate hexahydrate (Y(NO_3_)_3_·6H_2_O) (purity ~99.8%) as precursors. Equal volumes of deionised water (18.3 MΩ·cm resistance) and absolute ethanol were stirred at 353 ± 0.5 K. A solution of ZrOCl_2_·8H_2_O was prepared and added to the mixture. After the solution had re-stabilised at 353 ± 0.5 K, a stoichiometric amount of Y(NO_3_)_3_·6H_2_O was added to the solution (not before vacuum drying and the removal of excess water from highly hygroscopic yttrium nitrate). The solutions of the precursors were prepared with 7 and 8 mol % ratios of Y_2_O_3_ to (Y_2_O_3_ + ZrO_2_) for generation of the 7 YSZ and 8 YSZ compositions, respectively. The mixture was then heated at 353 K for 2 h, allowing hydrolysis to occur. Afterwards, an ammonium hydroxide solution (1.5 M) was dripped slowly into the mixture, which turned whiter and more viscous with each drop. Finally, the gel-point was observed clearly, and the dripping continued until the pH value was 9. The gel’s precipitates were repeatedly rinsed with a large amount of distilled water. The filtrate was tested with a silver nitrate solution (AgNO_3_). The absence of silver chloride (AgCl) precipitates (an insoluble compound) verified that no chlorides remained in the gel’s precipitates. Eventually, the wet precipitates were dried using two different drying techniques: freeze-drying and contact-drying on a simple heating plate. For the first, the gel was indirectly frozen by placing it in a closed beaker, which was submerged into liquid nitrogen and then freeze-dried for 3 days at 223 K in a vacuum chamber. The powder was then divided into different samples which were calcined in air using alumina (Al_2_O_3_) crucibles for 2 h at 353 K, 973 K and 1373 K.

The composition analysis of the sol-gel powder was investigated by Energy Dispersive X-ray Spectroscopy (EDS, HR-SEM; JSM-7400, JEOL, Tokyo, Japan). The crystalline phase composition was determined by X-ray diffraction analysis (XRD; DMAX 2100 powder diffractometer, Rigaku, Japan) and Transmission Electron Microscope (TEM; JEM-2010F, JEOL, Tokyo, Japan). Crystal size and pore size were approximated based on the measurement of more than 150 well-defined crystals and 150 well-defined voids using *ImageJ* (image processing software, National Institutes of Health, Bethesda, MD, USA).

## 3. Results

**Figure 1:**
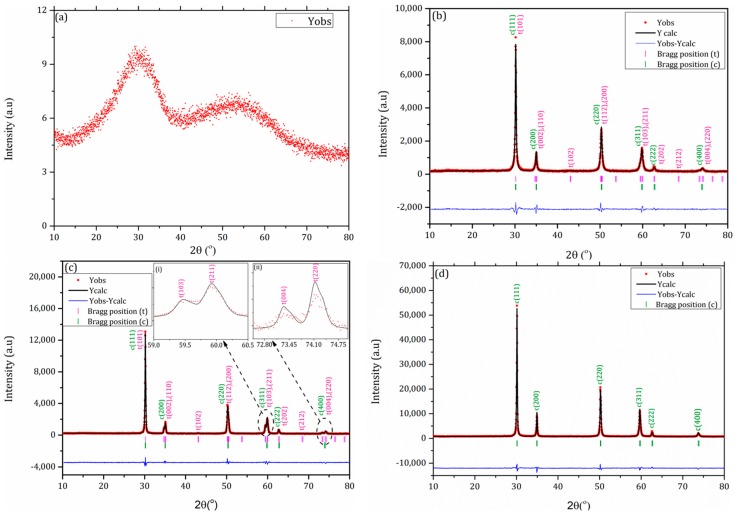
Rietveld refinements of the XRD patterns of 7 YSZ powder calcined for 2 h at: (**a**) 353 K, (**b**) 973 K, (**c**) 1373 K and of 8 YSZ powder calcined at (**d**) 1373 K for 2 h.

Figure 1 shows a graphical representation of the final Rietveld refinement, the experimental data and the difference between them for 7 YSZ and 8 YSZ powders. The reflections of 7 YSZ in the entire 2θ range of 20–80°, for powder calcined at 353, 973 and 1373 K for 2 h, are shown in Figure 1a–c, while those of 8 YSZ are shown in Figure 1d. Regarding the 8 YSZ composition, only single peaks of the cubic (*Fm*3*m*) phase reflections were observed for all tested calcination temperatures (the analysis for 1373 K was shown as a representative case). The reflections of the 7 YSZ composition are a combination of the tetragonal (*P*42/*nmc*) and the cubic (*Fm*3*m*) phases. The insert (i) in Figure 1c demonstrates a clear close-up at the (103) and (211) doublet, and the insert (ii) in Figure 1c shows a close up at the (004) and (220) doublet of the tetragonal phase. At 353 K, the lowest calcination temperature, the structure is quasi-amorphous, but at higher calcination temperatures of 973 and 1373 K, the structure is a mixture of the tetragonal (*P*42/*nmc*) and cubic (*Fm*3*m*) phases. Rietveld (FullProf) analysis showed that for the calcination temperatures of 973 and 1373 K, the amount of the tetragonal phase was 32% and 58%, respectively. It should be noted that no evidence of the undesired monoclinic (*P*2_1_/*c*) phase (the low temperature stable phase of pure ZrO_2_) was observed.

**Figure 2:**
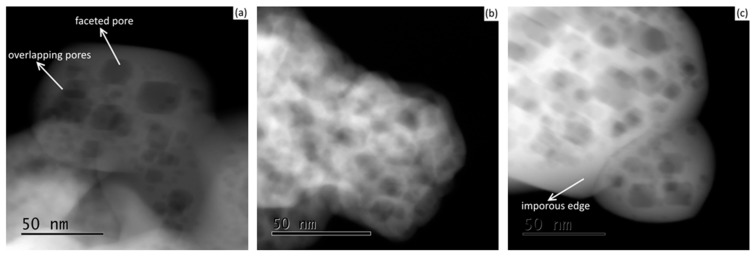
TEM images of 7 YSZ powder indicating YSZ particles containing pores: (**a**) before calcination; and after calcination at (**b**) 973 K, and (**c**) 1373 K for 2 h.

In terms of powder morphology, it can be seen that, following freeze-drying, internal nano-faceted pores (or voids) were present both before (Figure 2a) and after (Figure 2b,c) powder calcination at different temperatures of 973 and 1373 K.

**Figure 3:**
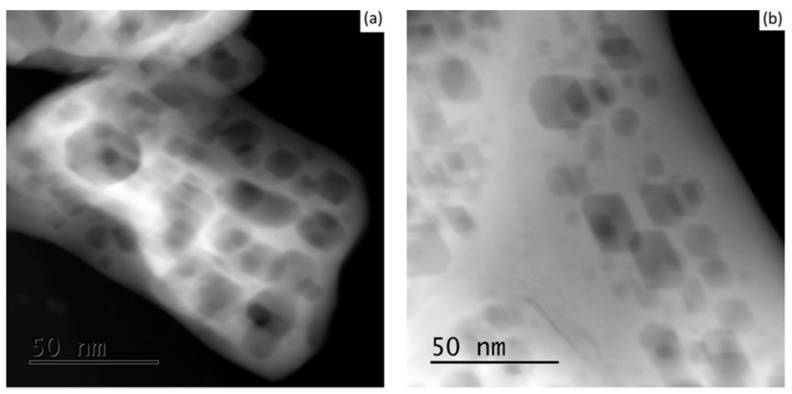
TEM images of 8 YSZ powder indicating YSZ particles containing pores after calcination at (**a**) 973 K and (**b**) 1373 K for 2 h.

Similar textures of faceted pores were observed at the different tested calcination temperatures; however, at the higher temperature of 1373 K, the particle’s edges were relatively more destitute of pores as an indication of the first stage of the sintering process. Moreover, no noticeable variations were observed between the 7 YSZ and 8 YSZ compositions in all cases. Representative cases are shown in Figure 3.

**Figure 4:**
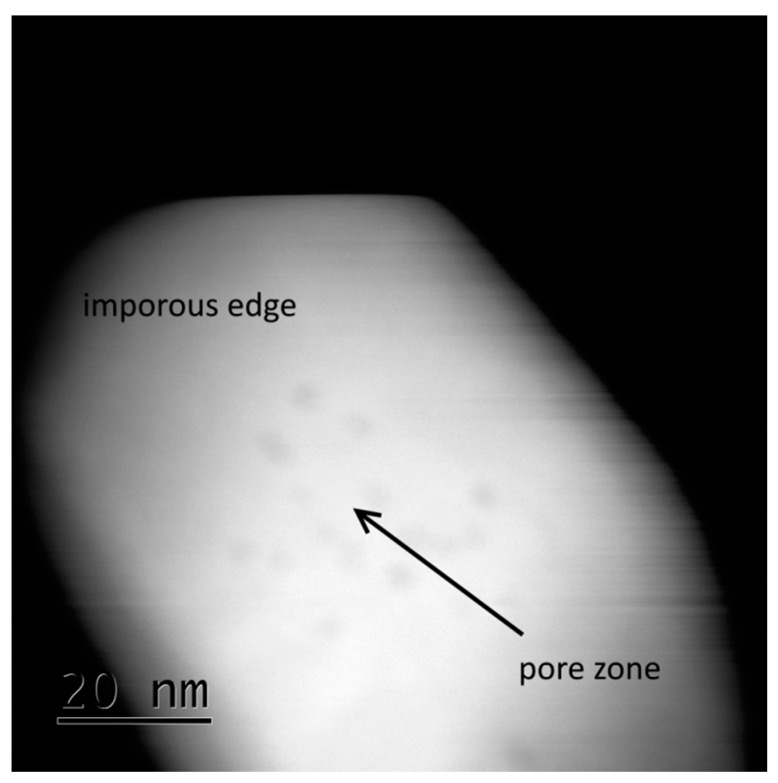
TEM images of 7 YSZ powder indicating YSZ particles containing small pores and smooth edges after calcination at 1373 K for 2 h.

In both compositions, before calcination and after calcination at 973 K, the average pore size was approximately 9 nm. According to a not-shown analysis of variance (ANOVA) with a significance level of 0.05, there is no statistically significant void size variation between before calcination and after calcination at 973 K (*p* value of 0.32). There is also no statistically significant difference in the mean void size between 7 YSZ powder and 8 YSZ powders (*p* value of 0.635). For a calcination temperature of 1373 K, smaller pores were difficult to measure, since their boundaries were not well defined, as demonstrated in Figure 4.

In the current research, the observation of the nano-voids both before and after calcination eliminates the possibility that pores were formed due to the release of residual synthesis by-products during calcination. In the case of pure monoclinic zirconia, the similar morphology of nano-particles containing pores has previously been observed via TEM following a different synthesis procedure, the sol-gel-hydrothermal method [19]. It was reported by Chang et al. that the gaseous hydrolysates, upon addition of superstoichiometric urotropine, were the origin of pores.

**Figure 5:**
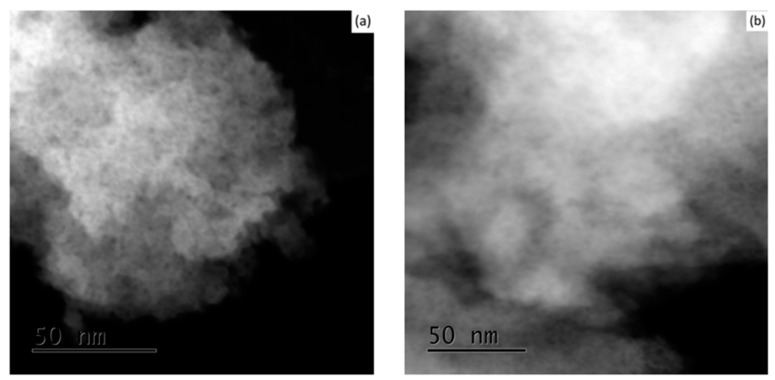
TEM images of 8 YSZ (**a**) and 7 YSZ (**b**) before calcination for contact drying method.

The relatively large size range of crystallites (25–200 nm), and the large crystal size before calcination (average size 80 nm), are not typical to the sol-gel method. This can be explained by the nucleation of pores inside each single crystal, enlarging its effective size, following the freeze-drying procedure. After verification that the calcination procedure does not contribute to the formation of the nano-voids, similar sol-gel synthesised YSZ samples were dried by standard contact drying for a comparison to the freeze-drying method, as can be seen by the TEM images in Figure 5a,b, respectively.

From these figures, it is clear that the drying technique has a major effect on the final powder morphology of YSZ. The porous structure did not occur when using the contact-drying technique. During contact drying, as evaporation empties the pores, solvent molecules diffuse towards the pores. The capillary forces created inside the pores apply compressive stresses that suck the gel’s network inwards and shrink it to form xerogel. However, during freeze-drying, the ice crystals inside the pores sublime directly as a gas phase, leaving empty pores in their place. Gas molecules have negligible intermolecular interactions, and therefore very low surface tension, which eliminates capillary stress. Similar internal nano-structures of hexagonal ice crystals have been shown elsewhere via TEM [20,21,22]. In our experiment, it is evident from Figure 2 and Figure 3 that, following calcination, the delicate nano-faceted pores in sol-gel synthesised YSZ powder were preserved.

## 4. Conclusions

In the current research, a sol-gel technique followed by a freeze-drying process is proposed for the first time for stabilising cubic (*Fm*3*m*) or mixed cubic (*Fm*3*m*)-tetragonal (*P*42/*nmc*) YSZ phases, depending on the composition and the calcination temperature, with internal nano-voids. XRD analysis showed that tetragonal crystal structure in 7 YSZ is favoured by increasing the calcination temperature up to 1373 K. It was found that the gel’s freeze-drying technique is responsible for the formation of the unique nano-voids. This technique is clean, simple and avoids any kind of organic additives.
